# Ferryl Hemoglobin Inhibits Osteoclastic Differentiation of Macrophages in Hemorrhaged Atherosclerotic Plaques

**DOI:** 10.1155/2020/3721383

**Published:** 2020-02-27

**Authors:** Erzsébet Zavaczki, Tamás Gáll, Abolfazl Zarjou, Zoltán Hendrik, László Potor, Csaba Zsigmond Tóth, Gábor Méhes, Ágnes Gyetvai, Anupam Agarwal, György Balla, József Balla

**Affiliations:** ^1^HAS-UD Vascular Biology and Myocardial Pathophysiology Research Group, Hungarian Academy of Sciences, Debrecen, Hungary; ^2^Department of Internal Medicine, Faculty of Medicine, University of Debrecen, Debrecen, Hungary; ^3^Department of Pediatrics, Faculty of Medicine, University of Debrecen, Debrecen, Hungary; ^4^Kálmán Laki Doctoral School of Biomedical and Clinical Sciences, University of Debrecen, Debrecen, Hungary; ^5^Nephrology Research and Training Center, Department of Medicine, University of Alabama at Birmingham, Birmingham, AL, USA; ^6^Department of Pathology, Faculty of Medicine, University of Debrecen, Debrecen, Hungary; ^7^Department of Vascular Surgery, Faculty of Medicine, University of Debrecen, Debrecen, Hungary

## Abstract

Intraplaque hemorrhage frequently occurs in atherosclerotic plaques resulting in cell-free hemoglobin, which is oxidized to ferryl hemoglobin (FHb) in the highly oxidative environment. Osteoclast-like cells (OLCs) derived from macrophages signify a counterbalance mechanism for calcium deposition in atherosclerosis. Our aim was to investigate whether oxidized hemoglobin alters osteoclast formation, thereby affecting calcium removal from mineralized atherosclerotic lesions. RANKL- (receptor activator of nuclear factor kappa-*Β* ligand-) induced osteoclastogenic differentiation and osteoclast activity of RAW264.7 cells were studied in response to oxidized hemoglobin via assessing bone resorption activity, expression of osteoclast-specific genes, and the activation of signalization pathways. OLCs in diseased human carotid arteries were assessed by immunohistochemistry. FHb, but not ferrohemoglobin, decreased bone resorption activity and inhibited osteoclast-specific gene expression (tartrate-resistant acid phosphatase, calcitonin receptor, and dendritic cell-specific transmembrane protein) induced by RANKL. In addition, FHb inhibited osteoclastogenic signaling pathways downstream of RANK (receptor activator of nuclear factor kappa-*Β*). It prevented the induction of TRAF6 (tumor necrosis factor (TNF) receptor-associated factor 6) and c-Fos, phosphorylation of p-38 and JNK (c-Jun N-terminal kinase), and nuclear translocation of NF*κ*B (nuclear factor kappa-*Β*) and NFATc1 (nuclear factor of activated T-cells, cytoplasmic 1). These effects were independent of heme oxygenase-1 demonstrated by knocking down HO-1 gene in RAW264.7 cells and in mice. Importantly, FHb competed with RANK for RANKL binding suggesting possible mechanisms by which FHb impairs osteoclastic differentiation. In diseased human carotid arteries, OLCs were abundantly present in calcified plaques and colocalized with regions of calcium deposition, while the number of these cells were lower in hemorrhagic lesions exhibiting accumulation of FHb despite calcium deposition. We conclude that FHb inhibits RANKL-induced osteoclastic differentiation of macrophages and suggest that accumulation of FHb in a calcified area of atherosclerotic lesion with hemorrhage retards the formation of OLCs potentially impairing calcium resorption.

## 1. Introduction

Cardiovascular disease is the leading cause of death worldwide [1] and vascular calcification is one of the independent risk factors associated with such morbidity and mortality [2–5]. Pathogenesis of vascular calcification is an active, finely tuned process with many similarities to the mechanism of skeletal bone formation [6]. In the bone, mineral deposition by osteoblasts and bone resorption by osteoclasts (OCs) are synchronized processes [7]. Disruption of the balance between osteoblast and OC activity may have pathological consequences such as osteoporosis or osteopetrosis [8, 9].

OCs are multinucleated cells derived from monocyte/macrophage lineage specialized for bone resorption [10]. The differentiation of macrophages to OCs requires macrophage/monocyte colony-stimulating factor-1 (M-CSF) and the receptor activator of nuclear factor kappa-*Β* ligand (RANKL) [11]. RANKL interacts with the receptor activator of nuclear factor kappa-B (RANK) activating downstream genes including tumor necrosis factor (TNF) receptor-associated factor 6 (TRAF6) [12], c-Jun N-terminal kinase (JNK) [13], p38 [14], nuclear factor-kappa B (NF*κ*B) [15], c-Fos [16], and nuclear factor of activated T-cells, cytoplasmic 1 (NFATc1) [17]. NFATc1 plays a pivotal role in OC maturation by regulating the expression of OC-specific genes, such as dendritic cell-specific transmembrane protein (DC-STAMP), tartrate-resistant acid phosphatase (TRAP), cathepsin K (CatK), and calcitonin receptor (CTR) [18–22].

RANKL and M-CSF are both expressed in calcifying vessels [23, 24]. In atherosclerotic plaques, calcification and bone formation are common phenomena characterized by the presence of vascular smooth muscle cells (VSMCs), osteoblasts, and osteoclast-like cells (OLCs) [25]. OLCs differentiate from infiltrating macrophages and colocalize with cholesterol deposition and mineralization [26]. An intriguing model has been raised by Doherty and coworkers suggesting that arterial calcium deposits represent a unique scenario which might favor the formation of OLCs from hematopoietic precursors possibly limiting calcification in atherosclerosis [27]. This hypothesis is further supported by the fact that Runx2, a key transcription factor that induces transition of VSMCs to osteoblast-like cells, directly binds to the promoter of RANKL and activates its expression leading to mineral deposition by VSMCs-derived osteoblasts and mineral resorption by OLCs [28]. Within the intramural compartment of the arteries, OLCs might degrade mineral deposits, thereby attenuating calcification and counterbalancing the activity of VSMC-derived osteoblasts [26]. The imbalance between bone formation by VSMC-derived osteoblasts and bone resorption by OLCs triggers pathological calcification process in the vessel walls.

Li and coworkers have described that complicated plaques with hemorrhage are characterized by a highly oxidative scenario creating a “death zone” for red blood cells (RBCs) [29]. RBCs in these death zones are lysed, and free Hb is subjected to rapid oxidation forming met-hemoglobin (MetHb, Fe^3+^) and ferryl hemoglobin (FHb, Fe^4+^ = O^2−^). Importantly, oxidation of Hb also leads to the release of heme moieties [30]. A significant body of evidence suggests that MetHb and FHb are present in hemorrhagic complicated plaques [31]. Our research group previously reported that FHb is a potent proinflammatory agonist in endothelial cells that induces morphological changes [32], increases monolayer permeability, and enhances monocyte adhesion [33]. These data suggest that oxidized Hb forms are involved in the pathogenesis of atherosclerosis.

The massive Hb content of hemorrhagic atheromas prompted us to examine whether the compensatory effect of OLCs in vascular calcification is influenced by products of Hb oxidation. The purpose of this study was to investigate the role of oxidized Hb in OC formation and resorption of calcium in calcified atheromas.

## 2. Materials and Methods

### 2.1. Study Approval

Carotid arteries were obtained from patients who underwent carotid endarterectomies from the Department of Surgery at the University of Debrecen. The collection was approved by the Scientific and Research Ethics Committee of the Scientific Council of Health of the Hungarian Government under the registration number of DE OEC RKEB/IKEB 3712-2012. Written informed consents were received from each participant. The study protocol conforms to the ethical guidelines of the 1975 Declaration of Helsinki.

### 2.2. Cell Culture and Reagents

Murine macrophage RAW264.7 cells were obtained from ATCC (Manassas, VA, USA). RANKL was purchased from Miltenyi Biotec (Bergisch Gladbach, Germany). Unless otherwise mentioned, all other reagents were obtained from Sigma (Saint Louis, MO, USA). Cells were cultured in DMEM containing 10% FBS, 100 U/mL penicillin, 100 *μ*g/mL streptomycin and neomycin, and 1 mM of sodium pyruvate (culture medium). Heme was dissolved in 20 mM NaOH.

### 2.3. *In vitro* Osteoclastogenesis

Cells (2 × 10^4^/cm^2^) were seeded onto 24-well plates and cultured in growth medium supplemented with RANKL (50 ng/mL) (osteoclastogenic medium) in the presence or absence of Hb (10 *μ*mol/L heme group), MetHb (10 *μ*mol/L heme group), and FHb (10 *μ*mol/L heme group) as indicated. The medium was changed every 2-3 days.

### 2.4. Immunohistochemistry

Carotid artery specimens were fixed with PBS formaldehyde (4%) solution at pH 7.4 for 1 to 3 days based on the size of the sample. After fixation, calcified samples were decalcified with 1.0 mol/L EDTA/Tris buffer. Paraffin-embedded 5-*μ*m-sections were deparaffanized in xylenes, rehydrated in a series of ethanol rinses from 100% to 70%, then washed with distilled water. Antigen retrieval was performed in RE7119 buffer (Leica, Wetzlar, Germany) at 95°C for 30 minutes. Sections were allowed to cool slowly, washed in distilled water, and incubated in 0.5% H_2_O_2_ for 10 minutes. For immunohistochemistry, samples were incubated with Dako EnVision FLEX Peroxidase-Blocking Reagent (Dako, Glostrup, Denmark) for 5 min in a wet chamber. Slides were then washed with EnVisionTM FLEX Wash Buffer, Tris-buffered saline solution containing Tween 20, and pH 7.6 (±0.1). Serial sections slides were incubated with antibodies against TRAcP (Roche, Mannheim, Germany, ready-to-use) or cathepsin K (Abcam, Cambridge, UK) at a dilution of 1 : 200, or CD68 (Roche, ready-to-use) or anti-FHb polyclonal antibody at a dilution of 1 : 50 using the ultraview universal DAB detection kit following the manufacturer's instructions. The intensity and distribution of TRAcP, cathepsin K, CD68, and FHb specific immunostaining were assessed by light microscopy (Leica DM2500 microscope, DFC 420 camera and Leica Application Suite V3 software, Leica). Samples were counterstained with Gill's Hematoxylin solution (105175 Merck Millipore, Billerica, MA, United States), rinsed in running tap water for 5 min and dehydrated through 95% ethanol for 1 min and 100% ethanol for 2 × 3 min followed by a clearing step in xylene for 2 × 5 min. The sections were then mounted using mounting media.

### 2.5. Isolation of Bone Marrow Monocytes

Bone marrow-derived monocytes (BMDMs) were isolated from wild type and heme oxygenase-1- (HO-1-) deficient mice (-/-) as described earlier [34]. BMDMs were treated for 5 days with RANKL (100 ng/mL) and MCS-F (50 ng/mL) in the presence and absence of FHb (10 *μ*mol/L).

### 2.6. Hemoglobin Preparation

Hb of different redox states, that is, (Fe^2+^) oxyHb, (Fe^3+^) MetHb, and (Fe^4+^) FHb, was prepared as described [32]. Briefly, Hb was isolated from fresh blood drawn from healthy volunteers using ion-exchange chromatography on a DEAE Sepharose CL-6B column. To generate MetHb, purified Hb was incubated with a 1.5-fold molar excess of K_3_Fe (CN)_6_ over heme for 30 min at 25°C. FHb was obtained by incubation of Hb for 1 h at 37°C with a 10 : 1 ratio of H_2_O_2_ to heme. Then, MetHb and FHb were dialyzed against saline (3 times for 3 hours at 4°C) and concentrated using Amicon Ultra centrifugal filter tubes (10,000 MWCO, Millipore Corp., Billerica, MA, USA). Aliquots were snap-frozen in liquid nitrogen and stored at −80°C. The purity of each Hb preparation was evaluated by SDS-PAGE followed by staining with the ProteoSilver Plus Silver Staining Kit. The purity of Hb preparations was above 99.9%. Hb concentrations were calculated as described by Winterbourn [35].

### 2.7. Spectral Scan of Human Carotid Arteries

Healthy carotid arteries, calcified atheromas, and calcified atheromas with hemorrhage were ground in liquid nitrogen, homogenized in phosphate-buffered saline (PBS, pH 7.4) followed by sonication on ice 3 times for 5 sec. Samples were then spinned at 12,000 × *g* for 20 minutes at 4°C, and the upper phase was measured by UV-visible spectra recorder (Beckman DU-800 spectrophotometer) from 500 nm to 700 nm wavelengths.

### 2.8. Tartrate-Resistant Acid Phosphatase (TRAP) Staining

For TRAP staining, cells were cultured in osteoclastogenic medium for 5 days. Osteoclastic differentiation was evaluated by TRAP staining using a leukocyte acid phosphatase kit according to the manufacturer's instructions. TRAP+multinucleated (more than 3 nuclei/cell) cells were identified as OCs. The areas of osteoclasts were measured by the ImageJ software.

### 2.9. Bone Resorption Assay

Bone resorptive activity of formed OCs was measured by bone resorption assay using Corning Osteo Assay Surface plate according to the manufacturer's instructions. The areas of resorption pit were determined with the ImageJ software.

### 2.10. Quantitative Reverse Transcription-Polymerase Chain Reaction

Total RNA was isolated using TRI Reagent (Zymo Research, Irvine, CA, USA), reverse transcribed with High-Capacity cDNA kit (Applied Biosystems, Foster City, CA), and real-time polymerase chain reactions were performed using fluorescent TaqMan probes. TaqMan gene expression assays for CTR (Mm00432282_m1), DC-STAMP (Mm04209236_m1), NFATc1 (Mm00479445_m1), HO-1 (Mm00516005_m1), RANK (Mm00437132_m1), and *β*-actin (Mm00607939_s1) were purchased from Thermo Scientific (Waltham, MA, USA). To measure the mRNA levels, the 20 *μ*L reaction mixture included 10 *μ*L of reverse transcribed sample (4 ng/*μ*L) and 10 *μ*L Master Mix (Thermo Scientific) containing 1 *μ*L TaqMan Assay (20×). PCRs were carried out using the iCycler iQ Real-Time PCR System (Bio-Rad Laboratories, Hercules, CA). Results were normalized by *β*-actin mRNA levels.

### 2.11. Cell Proliferation Assay

RAW264.7 cells were cultured in 24-well plates in growth medium and osteoclastogenic medium alone or supplemented with FHb for 1-4 days. The amount of viable cells was assessed by MTT assay.

### 2.12. Cytoplasmic and Nuclear Protein Extraction

Cells were grown in 6-well plates in growth medium or osteoclastogenic medium in the presence or absence of FHb for indicated time. Cells were washed three times with PBS and lysed with Harvest buffer (10 mM HEPES pH 7.9, 50 mM NaCl, 0.5 M sucrose, 0.5% Triton X-100, and protease inhibitors). After 10 min of incubation on ice, samples were spinned at 1000 × *g* for 5 min and supernatants were collected as cytosolic fraction. Pellets containing the nuclear fraction were washed three times with wash buffer (10 mM HEPES pH 7.9, 10 mM KCl, 0.1% NP-40, and protease inhibitors) and solubilized in nuclear protein extraction buffer (50 mM Tris pH 7.5, 150 mM NaCl, 0.5% sodium deoxycholate, 0.1% SDS, and protease inhibitors).

### 2.13. Immunofluorescence Staining

Cells were treated as described above with RANKL in the presence or absence of FHb. Cells were fixed in 4% paraformaldehyde in phosphate-buffered saline (PBS) pH 7.4 for 15 minutes. Coverslips were washed with PBS and samples were blocked with 5% goat serum in PBS supplemented with 0.3% Triton X-100 for 60 min. Samples were then incubated with primary antibody against NFATc1 (Novus Biologicals, Littleton, CO, USA) at a 1 : 250 dilution overnight at 4°C in antibody dilution buffer (1% BSA in PBS supplemented with 0.3% Triton X-100). The secondary antibody was a goat anti-mouse IgG conjugated to Alexa Fluor® 488 (Thermo Scientific) used at a 1 : 500 dilution in antibody dilution buffer and incubated for 60 min at room temperature. Nuclei were visualized with Hoechst. Nuclear translocation was investigated with TCS SP8 STED microscope using the Leica Application Software X (Leica, Mannheim, Germany).

### 2.14. Western Blot

HO-1, CTR, DC-STAMP, c-Fos, TRAF6, RANK expression, phosphorylation of p38, JNK, and cell-bound RANKL were analyzed by immunoblotting from whole-cell lysates with anti-HO-1 antibody (Proteintech, Manchester, UK) at 1 : 2000 dilution or anti-RANK antibody (Santa Cruz Biotechnologies, Santa Cruz, CA, USA) at 1 : 250 or anti-RANKL antibody (Abcam, Cambridge, UK) at 1 : 100 dilution or anti-CTR antibody (Proteintech) or anti-DC-STAMP antibody (Sigma) or anti-c-Fos antibody (Cell Signaling) or anti-TRAF6 antibody (Proteintech, Manchester, UK) or anti-phospho-p38 antibody (Cell Signaling, Danvers, MA, USA) or anti-p38 antibody (Cell Signaling) or anti-phospho-JNK antibody(Cell Signaling) or anti-JNK antibody(Cell Signaling) at 1 : 1000 dilution followed by HRP-labeled anti-mouse or anti-rabbit IgG antibody (Amersham Biosciences, Little Chalfont, UK). For CTR and DC-STAMP analysis, proteins were transferred with Dunn Carbonate Buffer. Nuclear translocation of NFATc1 and NF*κ*B were investigated using nuclear and cytosolic fractions with anti-NFATc1 antibody (Novus Biologicals) or anti-NF*κ*B antibody (Cell Signaling) at 1 : 500 dilution. Antigen-antibody complexes were visualized with the horseradish peroxidase chemiluminescence system (Amersham Biosciences). After detection, membranes were stripped and reprobed for GAPDH (Novus Biologicals) at 1 : 3000 or HSP90 (Cell Signaling) at 1 : 1000 or Lamin B1 (Proteintech) at 1 : 2000 dilution. Quantification of chemiluminesce was done by using the ImageJ software.

### 2.15. HO Activity Assay

Cells grown on 6-well plates were washed twice with Hank's Balanced Salt Solution (HBSS), and scraped and centrifuged at 2000 × *g* for 15 min at 4°C. Cells were re-suspended in 300 *μ*L of potassium phosphate (100 mmol/L (pH 7.4)) buffer containing 2 mmol/L MgCl_2_, frozen and thawed three times, and sonicated and centrifuged at 18,000 × g for 10 min at 4°C. Supernatants containing cell microsomes were used to measure HO activity as described previously [36]. HO activity is expressed as pmol bilirubin formed/mg cell protein per 60 min.

### 2.16. HO-1 Short-Interfering RNA (siRNA) Transfection

Small interfering RNA (siRNA) specific to HO-1 and negative control siRNA were obtained from Ambion (Austin, TX, USA). Transfection of siRNA into RAW264.7 cells was performed using the Oligofectamine Reagent (Invitrogen, Carlsbad, CA, USA). Briefly, cells were plated in antibiotic-free DMEM and cultured for 6 h. HO-1 siRNA at 40 nmol/L and transfection reagent complex were added to the cells in serum-free medium OptiMEM (Gibco, Thermo Scientific) for 16 h. Fresh normal growth medium was added then and the cells were incubated for another 8 h.

### 2.17. Detection of Crosslinked Hb by Western Blot

The detection of crosslinked Hb in three healthy carotid arteries, three atheromas, and three complicated carotid lesions with hemorrhage by Western blot was performed as described in our previous study [31] using HRP-conjugated goat anti-human Hb polyclonal antibody (ab19362-1 Abcam, Cambridge, UK) at a dilution of 1 : 15000.

### 2.18. Expression of Recombinant Mouse RANKL with 6× His Tag

For in vitro interaction assay, recombinant mouse RANKL with N-terminal 6 × His tag was expressed in E. coli Rosetta 2. Total mRNA was isolated from the lung tissue of C57BL mice, reverse transcribed, cloned into pTriex-4 Neo, and verified by sequencing. To express His-tagged RANKL, E. coli Rosetta 2 cells were transformed and cultured in Luria-Bertani medium containing 100 *μ*g/mL carbenicilllin at 30°C 250 rpm until OD600 = 0.5, then protein expression was induced with 1 mM Isopropyl *β*-D-1-thiogalactopyranoside (IPTG) followed by shaking at 250 rpm at 30°C for 3 h. Cells were then pelleted at 3990 × *g* at room temperature for 15 min and lysed with cold lysis buffer pH 8.0 (50 mM NaH_2_PO_4_, 300 mM NaCl, 1% Triton X-100, protease inhibitors, and 1 mg/mL lysozyme). His-tagged RANKL was purified using Protino Ni-TED 150 packed columns according to the manufacturer's guide. Endotoxin contamination was removed using Pierce™ High Capacity Endotoxin Removal Resin (Thermo). The quality of recombinant RANKL was analyzed by Coomassie staining and immunoblot. Recombinant His-tagged RANKL was effective to induce OC formation demonstrated by TRAP staining.

### 2.19. In Vitro RANK-RANKL Interaction Assay

To study the inhibitory effect of FHb on RANK-RANKL interaction in a test tube experiment, RANK was purified from RAW cells using immunoprecipitation. Briefly, cells were lysed with cold lysis buffer containing 50 mM Tris pH 7.5, 150 mM NaCl, 2× protease inhibitor cocktail, and 1% Triton X-100; incubated on ice for 10 min; and clarified by centrifugation at 14000 × g for 10 min at 4°C. Supernatants were then gently rocked at 4°C with 15 *μ*g of RANK antibody overnight, then antigen-antibody complexes were coincubated with pre-washed protein A/G magnetic beads (Thermo Scientific) for 60 min at room temperature. Beads were then washed three times with cold wash buffer containing detergent (50 mM Tris pH 7.5, 150 mM NaCl, 0.05% Igepal CA630, and protease inhibitors), then three times with wash buffer without detergent (50 mM Tris pH 7.5, 150 mM NaCl), and coincubated with 1 *μ*g RANKL or 1 *μ*g RANKL and 10 *μ*M FHb at room temperature for 60 min. Beads were then washed three times with 50 mM Tris pH 7.5 and 150 mM NaCl, and samples were eluted with 2 × SDS sample buffer without reducing agent at 50°C for 10 min, supplemented with 100 mM DTT and subjected to immunoblot analysis.

### 2.20. Statistical Analysis

Statistical analysis was performed with GraphPad Prism 5 by one-way ANOVA test followed by post hoc Bonferroni's Multiple Comparison test or *t* test. A significant value of *p* < 0.05 was marked with ^∗^, *p* < 0.01 with ^∗∗^, and *p* < 0.001 was marked with ^∗∗∗^. Nonsignificant (ns) differences were also marked. Data are shown as mean ± SEM.

## 3. Results

### 3.1. FHb Inhibits Osteoclastogenesis and OC Bone Resorption Activity

**Figure 1:**
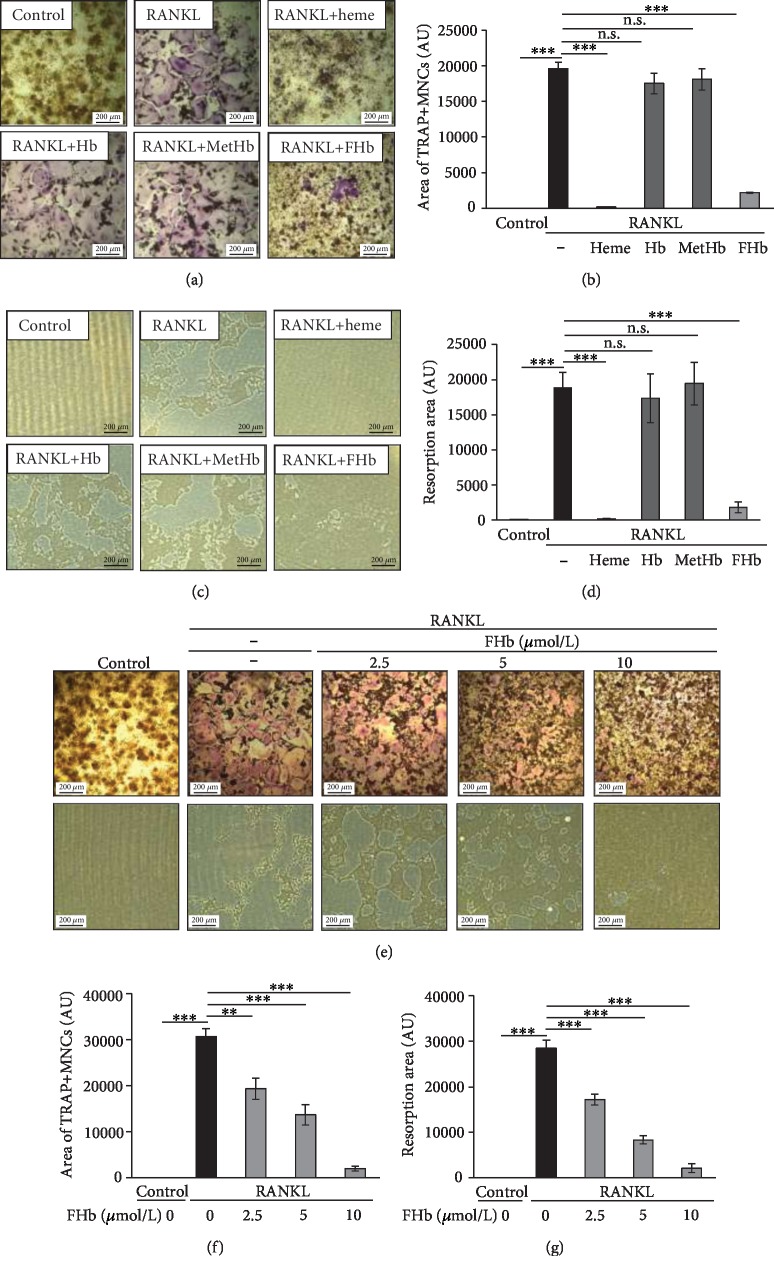
FHb inhibits osteoclastogenesis and OCs bone resorption activity in vitro (a) RAW264.7 cells were cultured on 24-well plates in control growth medium or in osteoclastogenic medium (control growth medium supplemented with 50 ng/mL RANKL) or in osteoclastogenic medium with heme (50 *μ*mol/L), Hb (10 *μ*mol/L heme group), MetHb (10 *μ*mol/L heme group), or FHb (10 *μ*mol/L heme group) for 5 days and stained for tartrate-resistant acid phosphatase (TRAP). Representative images of stained microscopic views (magnification 100×) from three independent experiments are shown. (b) Areas of TRAP-positive multinucleated cells were determined in ten microscopic field areas and compared with untreated cells. Data are expressed as mean ± SE of three independent experiments. (c) RAW264.7 cells were cultured on Osteo-Assay Surface plate in control growth medium or in osteoclastogenic medium (control growth medium supplemented with 50 ng/mL RANKL) or in osteclastogenic medium with heme (50 *μ*mol/L), Hb (10 *μ*mol/L heme group), MetHb (10 *μ*mol/L heme group), or FHb (10 *μ*mol/L heme group) for 6 days. Representative microscopic pictures of resorbed pits are shown (magnification 100×). (d) The areas of resorption pits were determined in ten microscopic field areas and compared with untreated cells. Data are expressed as mean ± SE of three independent experiments. (e) RAW264.7 cells were cultured on 24-well plates in control growth medium or in osteoclastogenic medium in the absence or presence of FHb (2.5, 5, and 10 *μ*mol/L heme group) for 5 days for tartrate-resistant acid phosphatase (TRAP) staining (upper panel) or on Osteo-Assay Surface plates for bone resorption assay (lower panel). Representative images of stained microscopic views (magnification 100×) from three independent experiments are shown. The areas of TRAP positive cells (f) or resorption pits (g) were determined in ten microscopic field areas and compared to untreated cells. Data are expressed as mean ± SE of three independent experiments.

Macrophages can transform into OCs in response to RANKL [37, 38]. To investigate the effect of Hbs on RANKL-induced osteoclastogenesis, murine macrophage RAW264.7 cells were cultured in osteoclastogenic medium containing 50 ng/mL RANKL in the presence or absence of Hb, MetHb, or FHb. First, we analyzed the effect of the different Hb forms on RANKL-induced osteoclastogenesis using TRAP staining (Figure 1(a)). Heme (50 *μ*mol/L), a potent inhibitor of RANKL-induced osteoclastogenesis, was used as positive control for inhibition assays. RANKL-induced OC formation was significantly inhibited either by heme (50 *μ*mol/L) or FHb (10 *μ*mol/L heme group), but not by Hb (10 *μ*mol/L heme group) or MetHb (10 *μ*mol/L heme group). To quantify OC formation, the areas of TRAP-positive cells were measured by the ImageJ software (Figure 1(b)). This analysis revealed that RANKL-induced OC formation was significantly (*p* < 0.001) impaired either by heme or FHb, but not by Hb or MetHb (Figure 1(b)). Next, we examined whether FHb inhibits bone resorption activity of OCs (Figure 1(c)). Our data suggest that both FHb and heme significantly (*p* < 0.001) reduced the resorption area of OCs, while neither Hb nor MetHb influenced bone resorptive activity (Figures 1(c) and 1(d)). Next, we examined whether a dose-response relationship exists between FHb concentration and inhibition of OC differentiation. We showed that FHb inhibited osteoclastogenesis in a dose-dependent manner, and as low as 2.5 *μ*mol/L FHb significantly prevented OC formation as evidenced by TRAP staining (Figure 1(e) upper panel and Figure 1(f)) and bone resorption assay (Figure 1(e) lower panel and Figure 1(g)). To explore whether this inhibitory effect of FHb is associated with its potential cytotoxic effect, we cultured RAW264.7 cells for 4 days in the presence or absence of RANKL and FHb. Cell proliferation and viability were analyzed with MTT assay at various time points (one to four days). Our results showed the proliferation of RAW264.7 cells decreased in response to RANKL as well as to RANKL+FHb compared with untreated cells; however, FHb did not affect proliferation compared with RANKL-treated cells (Supplementary fig. [Supplementary-material supplementary-material-1]). Next, we explored whether such decreased proliferation was associated with apoptotic cell death. Accordingly, we analyzed caspase-3 cleavage as a marker of apoptosis by immunoblot which showed that apoptotic cell death did not occur during OC differentiation in response to RANKL and FHb (Supplementary fig. [Supplementary-material supplementary-material-1]).

### 3.2. FHb Downregulates OC-Specific Gene Expression in Response to RANKL

**Figure 2:**
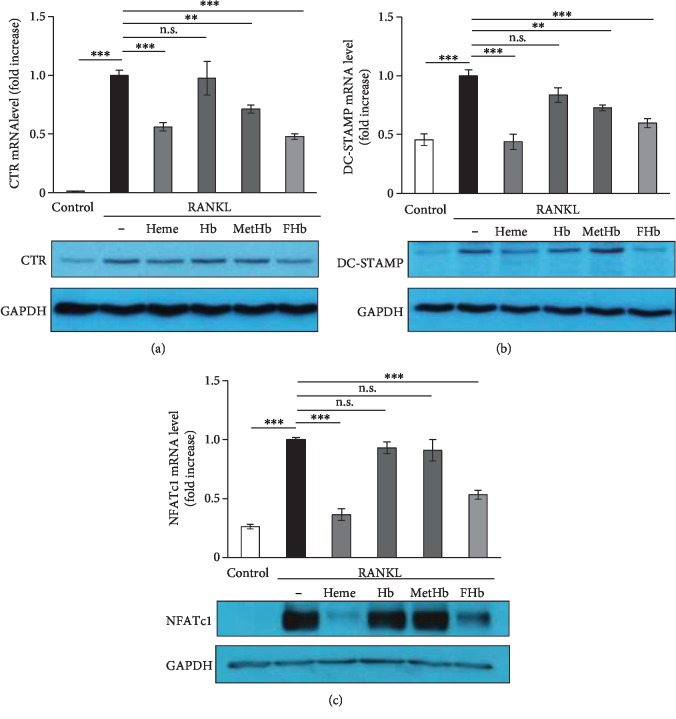
FHb downregulates OC-specific gene expression in response to RANKL RAW264.7 cells were cultured on 24-well plates in control growth medium or in osteoclastogenic medium (control growth medium supplemented with 50 ng/mL RANKL) or in osteoclastogenic medium with heme (50 *μ*mol/L), Hb (10 *μ*mol/L heme group), MetHb (10 *μ*mol/L heme group), or FHb (10 *μ*mol/L heme group). CTR (a), DC-STAMP (b), and NFATc1 (c) gene expressions were analyzed with quantitative RT-PCR and immunoblot after 5 days for CTR, 4 days for DC-STAMP, and 3 days for NFATc1. qRT-PCR was normalized to *β*-actin while immunoblot was normalized to GAPDH. Data are expressed as mean ± SE of three independent experiments.

Osteoclastic differentiation of macrophages requires changes in protein expression [39]; therefore, we examined the effect of various Hb forms on the expression of osteoclast-specific markers (CTR, DC-STAMP, and NFATc1) using quantitative real-time PCR (qRT-PCR) and immunoblotting. As expected, RANKL significantly upregulated CTR (Figure 2(a)) and DC-STAMP (Figure 2(b)) expression in RAW264.7 cells that was inhibited both by FHb (*p* < 0.001) and heme (*p* < 0.001) and, to a lesser extent, by MetHb (*p* < 0.01). Importantly, Hb did not prevent RANKL-induced CTR and DC-STAMP induction. Significantly, FHb and heme but not Hb and MetHb blunted NFATc1 (*p* < 0.001) expression induced by RANKL (Figure 2(c)).

### 3.3. Inhibitory Effect of FHb on Osteoclastogenesis Is Independent of HO-1

**Figure 3:**
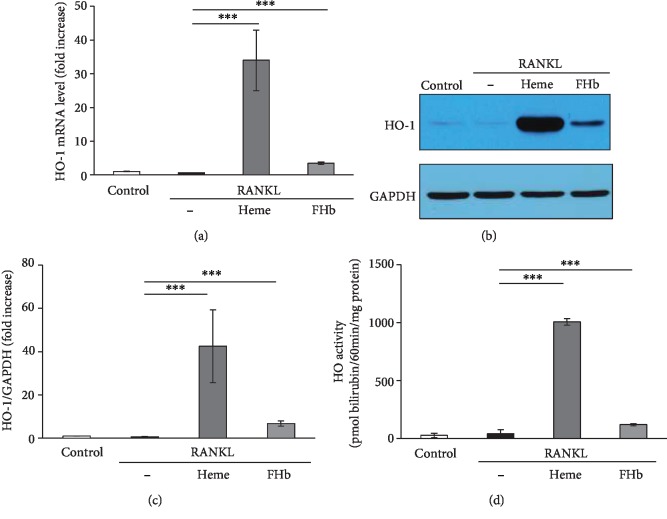
FHb induces HO-1 expression in RAW264.7 cells. RAW264.7 cells were grown on 24-well plates in control growth media or in osteoclastogenic media (control growth medium supplemented with 50 ng/mL RANKL) in the absence or presence of heme (50 *μ*mol/L) or FHb (10 *μ*mol/L heme group) for 5 days. (a) HO-1 mRNA levels were determined by quantitative RT-PCR and normalized to *β*-actin. Results are presented as mean ± SE of three independent experiments. (b–c) HO-1 protein expression was detected by Western blot and normalized to GAPDH after densitometric analysis. A representative image of three independent experiments is shown. (d) HO enzyme activity was measured as described in Section 2. Results are shown as mean ± SE from three independent experiments.

We have previously described that FHb induces HO-1 in endothelial cells [33] and it has been reported that upregulation of HO-1 by heme inhibits OC formation and bone resorption *in vitro* [40]. Therefore, we tested whether HO-1 mediates the inhibitory effect of FHb on OC formation. As shown in Figure 3, FHb, similar to heme, significantly induced HO-1 expression both at mRNA and protein levels (Figures 3(a)–3(c)) and increased HO enzyme activity (Figure 3(d)) in RAW264.7 cells.

**Figure 4:**
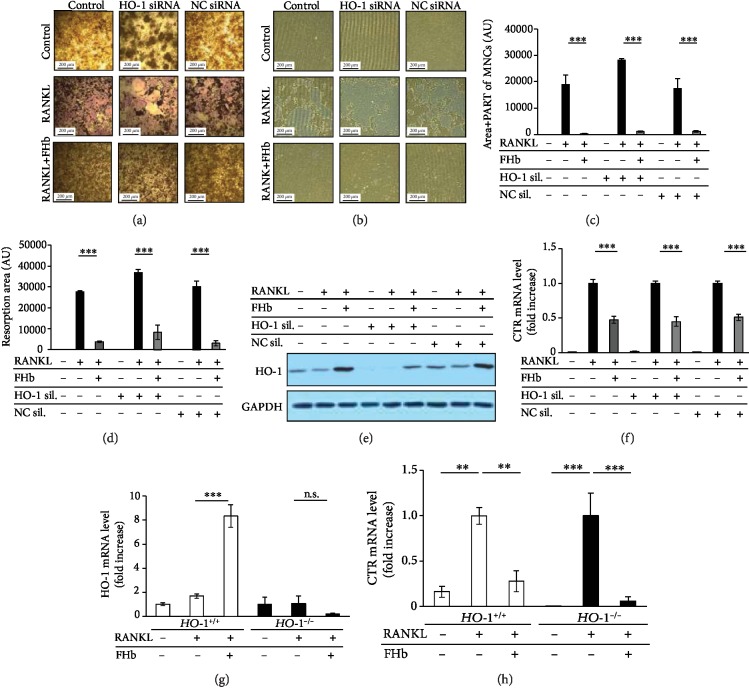
Inhibitory effect FHb on osteoclastogenesis is independent of HO-1. RAW264.7 cells were transfected with HO-1 or negative control siRNA (NC) and maintained in control growth medium or osteoclastogenic media (control growth medium supplemented with 50 ng/mL RANKL) in the absence or presence of FHb (10 *μ*mol/L heme group). OC formation in wild type, NC siRNA-treated, or HO-1-knocked down cells were analyzed by TRAP staining (a) or pit formation assay (b). The areas of TRAP positive cells (c) or resorption pits (d) were determined in ten microscopic field areas and compared with untreated cells. Data are expressed as mean ± SE of three independent experiments. (e) HO-1 protein expression was analyzed by immunoblot and normalized to GAPDH. A representative image of three independent experiments is shown. (f) CTR mRNA levels were measured by RT-PCR after 4 days and normalized to *β*-actin. Results are shown as mean ± SE from three independent experiments. (g, h) Bone marrow-derived macrophages (BMDMs) were isolated from wild type (*HO-1*^+/+^) and HO-1 knock down (*HO-1*^−/−^) mice and grown in culture medium with 50 ng/mL M-CSF or in osteoclastogenic medium (50 ng/mL M‐CSF + 100 ng/mL RANKL) in the presence or absence of FHb (10 *μ*mol/L heme group) for 5 days. RNA levels of HO-1 (g) and CTR (h) were measured by RT-PCR and normalized to *β*-actin. Results are shown as mean ± SE from three independent experiments.

We knocked down HO-1 expression by HO-1-specific siRNA and analyzed osteoclastogenesis in response to RANKL and FHb. Surprisingly, FHb prevented RANKL-induced OC formation in HO-1-silenced cells similar to the controls that was demonstrated by TRAP staining (Figures 4(a) and 4(c)) and bone resorption assay (Figures 4(b) and 4(d)). Silencing HO-1 expression was confirmed by immunoblotting (Figure 4(e)). To further verify our results at the molecular level, CTR expression—as OC marker—was monitored by q-RT-PCR in HO-1-knocked down cells after RANKL treatment. We showed that irrespective of the degree of HO-1 expression, administration of FHb significantly decreased RANKL-mediated CTR mRNA expression (Figure 4(f)).

We also assessed the ability of FHb to mitigate osteoclastogenesis in BMDMs obtained from wild-type (*HO-1*^+/+^) and HO-1 knock out (*HO-1*^−/−^) mice (*n* = 4). FHb significantly induced HO-1 mRNA expression in BMDMs derived from *HO-1*^+/+^ mice; however, no HO-1 expression was detected in BMDMs isolated from HO-1 knock out *HO-1*^−/−^ mice (Figure 4(g)). Importantly, FHb inhibited RANKL-induced CTR expression in both *HO-1*^+/+^ and in *HO-1*^−/−^ BMDMs (Figure 4(h)). These results support that the inhibitory effect of FHb on osteoclastogenesis is independent of HO-1 expression.

### 3.4. FHb Blocks RANK-RANKL Interaction

**Figure 5:**
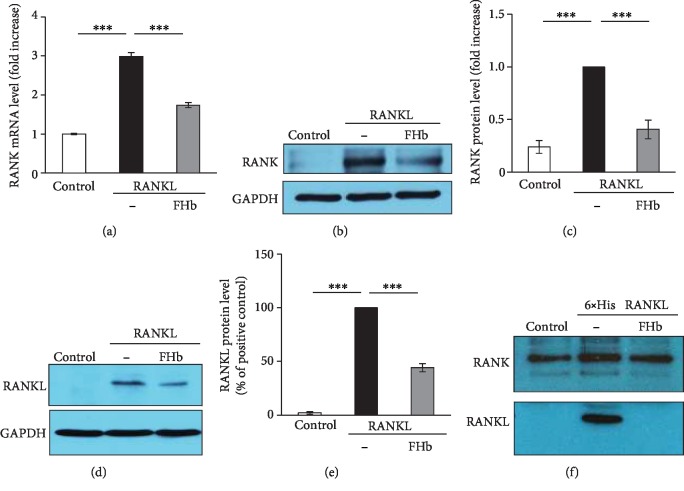
FHb inhibits RANK-RANKL interaction. RAW264.7 cells were grown in control growth media or in osteoclastogenic media (control growth medium supplemented with 50 ng/mL RANKL) in the absence or presence of FHb (10 *μ*mol/L heme group) for 3 or 5 days (a–e). (a) RANK mRNA levels were measured after 3 days by qRT-PCR and normalized to *β*-actin. Results are shown as mean ± SE from three independent experiments. (b) RANK protein expression was analyzed by immunoblot after 3 days and normalized to GAPDH. A representative image from four independent experiments is shown. (c) Densitometric analysis of RANK immunoblots is shown as mean ± SE from four independent experiments. (d) Cell association of RANKL was analyzed after 5 days by immunoblot specific to RANKL and normalized to GAPDH. (e) Densitometric analysis of RANKL immunoblots is shown as mean ± SE from three independent experiments. (f) RANK was immunoprecipitated from cell lysates and coincubated with 6 × His RANKL (1 *μ*g) in the presence or absence of FHb (10 *μ*mol/L heme group) for 1 hr. RANK-RANKL interaction was analyzed by immunoblot.

The formation of OCs requires RANKL attachment to its receptor RANK [41]. In addition, RANKL directly induces RANK expression [42]. To decipher the molecular mechanism by which FHb inhibits OC formation, we tested whether FHb influences RANKL-induced RANK expression. We demonstrated that FHb significantly attenuated RANK expression in response to RANKL both at mRNA (Figure 5(a)) and protein levels (Figures 5(b) and 5(c)). To gain a more mechanistic insight into the inhibitory effect of FHb on OC formation, we analyzed whether FHb inhibits the RANK-RANKL interaction by measuring the amount of cell-associated RANKL by immunoblot. We showed that the association of exogenous RANKL to cells was markedly decreased when FHb was present in the experimental medium (Figures 5(d) and 5(e)). To further verify this observation, we developed a recombinant His-tagged RANKL to study RANK-RANKL interaction in test tube experiments. The purity of our in-house His-tagged RANKL was validated by Coomassie staining and immunoblot (Supplementary fig. [Supplementary-material supplementary-material-1]). We demonstrated that His-tagged RANKL effectively induced OC formation in RAW cultures demonstrated by TRAP staining (Supplementary fig. [Supplementary-material supplementary-material-1]). In the test tube experiments, RANK was immunoprecipitated from RAW264.7 cell lysates and coincubated with His-tagged recombinant RANKL (1 *μ*g) in the presence or absence of FHb. The association of recombinant RANKL with RANK was analyzed by immunoblot. Test tube experiments corroborated our findings in cell cultures that FHb inhibited RANK-RANKL interaction (Figure 5(f)). These data suggest that the inhibitory effect of FHb on RANK expression and osteoclastic transformation of RAW264.7 cells were mediated by inhibition of the direct interaction between RANK and RANKL.

### 3.5. FHb Inhibits RANKL-Induced Signaling Involved in OC Differentiation

**Figure 6:**
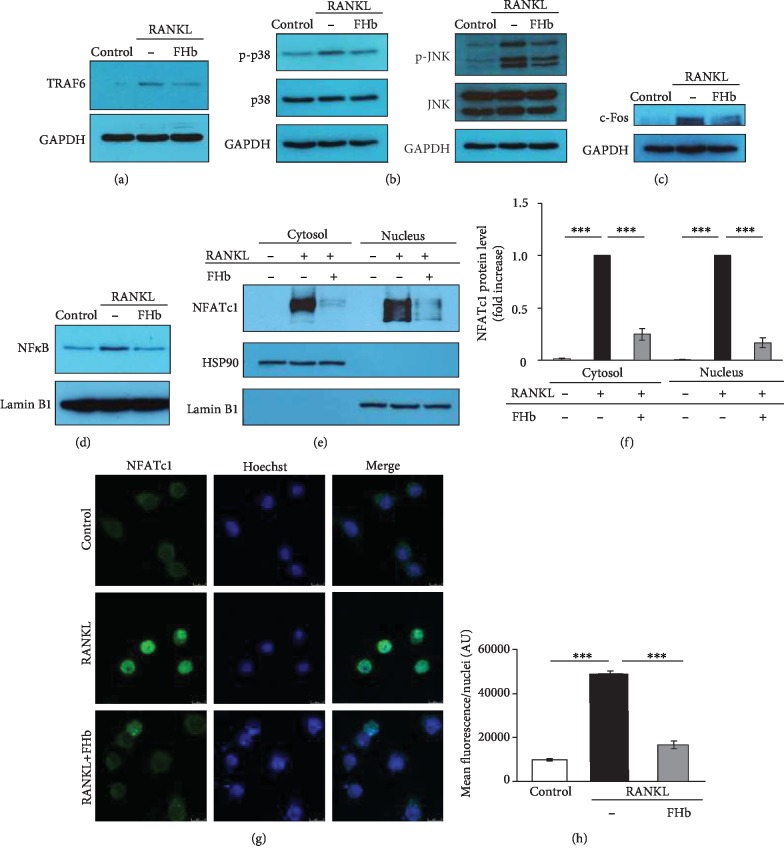
FHb inhibits RANKL-induced signaling pathways involved in OC differentiation. RAW264.7 cells were cultured on 24-well plates (for immunofluorescent staining) or 6-well plates (for immunoblot) in control growth media or in osteoclastogenic media (control growth media supplemented with 50 ng/mL RANKL) with or without FHb (10 *μ*mol/L). (a) TRAF-6 expression was analyzed by immunoblot after 2 days. (b) p38 and JNK phosphorylation were assessed at 40 min and normalized to p38, JNK, and GAPDH. (c) c-Fos activation was analyzed after 40 min and normalized to GAPDH. (d) Nuclear translocation of NF*κ*B was analyzed after 40 min from isolated nuclei and normalized to Lamin B1. (e, f) NFATc1 expression and nuclear translocation were analyzed by immunoblot and normalized to HSP90 (cytosolic protein) and Lamin B1 (nuclear protein). (g, h) NFATc1 expression and nuclear translocation were analyzed by immunofluorescence after 24 hrs. Results are shown as mean ± SE from three independent experiments.

RANK-RANKL interaction initiates a series of signaling events leading to OC formation from macrophages [43]. This includes TRAF6 which activates downstream pathways, such as NF*κ*B, JNK, p38, c-Fos, and NFATc1, which are all crucial factors in OC differentiation. Here, we showed that RANKL induced TRAF6 expression (Figure 6(a)), p38 and JNK activation (Figure 6(b)), c-Fos expression (Figure 6(c)), nuclear translocation of NF*κ*B (Figure 6(d)), and NFATc1 (Figures 6(e)–6(h)). Importantly, the exposure of cells to FHb prevented the induction of TRAF6 and c-Fos, phosphorylation of p38 and JNK, and nuclear translocation of NF*κ*B and NFATc1. These results corroborate our hypothesis that FHb inhibits RANK-RANKL interaction and its subsequent signaling pathways, thereby preventing OC differentiation from macrophages.

### 3.6. Oxidation of Hb Occurs in Calcified Lesions with Hemorrhage

**Figure 7:**
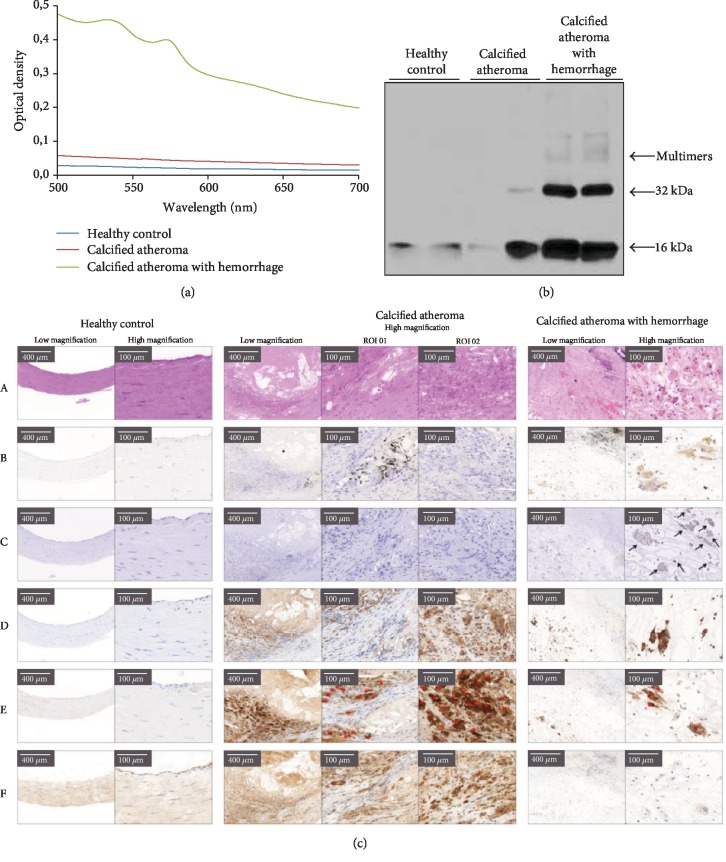
FHb is present in hemorrhagic calcified arteries and impairs OLCs formation. (a) Representative spectral scan of human tissue specimens derived from healthy carotid arteries, calcified atheromas, and calcified atheromas with hemorrhage. Spectophotometric determination of oxidized Hb was measured from 500 to 700 nm wavelengths. (b) Representative immunoblot (one out of three performed) demonstrates Hb oxidation in human vessels from two healthy arteries, two calcified atheromas, and two calcified atheromas with hemorrhage (20 *μ*g/lane). (c) Histological pattern analysis of healthy carotid arteries (left), calcified plaques (middle), and calcified lesions with hemorrhage (right). Basic histological architecture and cellular components are stained with H&E staining showing extravascular red blood cells in the calcified atheroma with hemorrhage (row A). Calcium deposits are visualized by Von Kossa staining (row B). The presence of FHb is showed by immunohistochemistry (row C). CD68-positive multinucleated giant cells (row D) were specified as OLCs with immunohistochemical staining specific to OC marker TRAP (row E) and cathepsin K (row F).

Our previous studies revealed that complicated lesions with hemorrhage contain oxidized forms of hemoglobin [31] which is also corroborated by our current study. Spectrophotometric analysis of the human vessel samples showed that oxidized Hb was present in the calcified atheromas with hemorrhage, while healthy arteries and calcified lesions did not contain oxidized Hb (Figure 7(a)). We observed the marked accumulation of crosslinked Hb dimers, tetramers, and multimers in hemorrhagic calcified plaques reflecting that Hb oxidation are extensive in these lesions compared with calcified atheromas without hemorrhage or healthy carotid arteries (Figure 7(b)).

### 3.7. Lack of OLCs Is Associated with the Presence of FHb in Hemorrhagic Calcified Lesions in Human Vessels

To examine whether FHb inhibits OLC formation in patients who underwent carotid endarterectomy, the presence of OLCs was analyzed in healthy carotid arteries, calcified atheromas, and calcified atheromas with hemorrhage (Figure 7(c)). Extracellular calcium deposits were present in calcified atheromas and in calcified atheromas with hemorrhage as evidenced by Von Kossa staining (row B). The presence of FHb was prominent in calcified atheromas with hemorrhage while no positive staining pattern could be seen in carotid arteries from healthy individuals or calcified lesions without hemorrhage (row C). Furthermore, multiple CD68 positive, multinucleated giant cells were detected in calcified lesions while the number of these cells was rather limited in calcified plaques with hemorrhage (row D). CD68 positive, multinucleated giant cells were not present in healthy carotid arteries (row D). Multinucleated giant cells showed the characteristics of OCs demonstrated by TRAP (row E) and CatK (row F) staining. A number of OLCs positive to TRAP and CatK staining were identified and were evident around the calcified area in calcified atheromas suggesting the presence of OLCs in calcified vessels. In sharp contrast, the number of OLCs in calcified plaques with hemorrhage was scarce despite the extensive calcification. These data demonstrate that the presence of OLCs in calcified atheromas with hemorrhage is significantly limited when compared to calcified lesions without hemorrhage.

## 4. Discussion

The presence of OLCs in the calcified area of atherosclerotic plaques is well demonstrated [44]. According to the “osteoclast theory,” an increased osteoblastic and a reduced osteoclastic activity might contribute to intima calcification [45]. Therefore, disturbances in OLC differentiation and activity in the atherosclerotic plaques might be considered a pathogenic factor in vessel wall calcification.

Vascular calcification is associated with increased cardiovascular morbidity and about one-fifth of the calcified vessels and valves contain trabecular bone [46]. The molecular mechanism of arterial calcification resembles bone mineralization sharing a number of similarities [47]. Under homeostatic conditions in bones, mineral deposition by osteoblast and resorption mediated by OCs are strictly coupled resulting in a delicate balance between bone anabolism and catabolism [48, 49].

The importance of functional OLCs in vascular calcification has been documented by several studies. Mice lacking carbonic anhydrase II, which is essential for bone resorption activity of OLCs, develop arterial calcification [50]. In addition, the lack of M-CSF, which is necessary for OLC formation, promotes vascular calcification in mice possibly due to the impaired OLC formation [51]. Overall, these findings suggest that functional OLCs are essential for mineral resorption in the vasculature, and that lack of OLC activity promotes vascular calcification. Multinucleated, TRAP-positive cells with typical osteoclastic morphology were identified in atherosclerotic lesions close to the mineralized areas [26]. This observation was also supported by our present results in human atherosclerotic carotid arteries demonstrating the presence of a vast number of OLCs with strong cathepsin K and TRAP positivity in the vessel wall areas where mineralization occurred. However, the number of OLCs was limited in the calcified atheromas with hemorrhage that were characterized by the presence of oxidized Hbs, and this observation supports our hypothesis that FHb abolishes OLCs from hemorrhagic, calcified carotid tissues resulting in insufficient mineral resorption from the vessel wall.

The formation of OLCs in the vasculature is dependent on RANKL that is abundantly secreted by a number of cell types, such as VSMCs and endothelial cells [52–54]. Following interaction with RANK, RANKL induces the activation of downstream signalization, such as tumor necrosis factor (TNF) receptor-associated factor 6 (TRAF6) [12], c-Jun N-terminal kinase (JNK) [13], p38 [14], receptor activator of nuclear factor-kappa B (NF*κ*B) [15], c-Fos [16], and NFATc1. NFATc1 activates a number of downstream genes such as CTR, cathepsin K, and TRAP [55]. The pivotal function of NFATc1 is highlighted by the significant impairment of osteoclastogenesis in OC-specific conditional NFATc1-deficient mice [56]. In addition, NFATc1-deficient embryonic stem cells are unable to differentiate into OCs in response to RANKL [55]. In our study, we showed that FHb but not MetHb or ferrous Hb inhibited the expression of NFATc1. In addition, the expression of other OC markers, such as CTR and DC-STAMP, was also alleviated by FHb, and, to a lesser degree, by MetHb, but not by ferrous Hb. These data indicate that the inhibitory effect of FHb on osteoclastogenesis is mediated by the suppression of the key genes involved in this process. Furthermore, we demonstrated that this inhibitory effect of FHb was dose-dependent, occurred even at low micromolar concentrations and was not associated with cell death. To explore the signaling events downstream of RANK in response to RANKL and FHb, we analyzed the key proteins involved in osteoclastogenesis. Previous studies have revealed that TRAF6 is essential in the cytoskeletal organization and resorptive activity of OC [12]. Mitogen-activated protein kinases (MAPKs), such as JNK and p38, are critical for normal osteoclastogenic differentiation and activation [13, 14]. NF*κ*B, together with c-Fos, is also important determinants of OC formation [15, 16]. Here, we showed that FHb blunted early signaling events of osteoclastogenesis involving MAPK and NF*κ*B activation as well as c-Fos and TRAF-6, thereby blocking the osteoclastogenic reprogramming of macrophages.

Previously, it was demonstrated that heme inhibits osteoclastogenesis as well as the expression of OC marker genes such as TRAP, CTR, and DC-STAMP *via* the induction of HO-1, a key enzyme of heme catabolism [40]. We have reported that MetHb but not ferrous Hb releases its heme prosthetic group that is taken up by endothelial cells followed by the activation of HO-1 [57]. Here, we demonstrated that FHb induced catalytically active HO-1 supposing that its heme group could be responsible for the inhibition of osteoclastogenesis [40]. Furthermore, others have demonstrated that deltamethrin, a pyrethroid pesticide or magnolol, an extract with anti-inflammatory properties isolated from *Magnolia officinalis* prevents OC formation in a HO-1 dependent fashion [58, 59]. These observations underscore the inhibitory effects of HO-1 in osteoclastogenesis. However, our results presented here showed that the inhibitory effect of FHb on OC formation was independent of HO-1. This notion was also corroborated by using murine BMDMs derived from *HO-1*^−/−^ that indicated other mechanisms are involved in FHb-mediated inhibition of OC formation that are independent of HO enzyme activity.

RANK and RANKL interaction is essential for OC formation, and RANK expression is vital for the differentiation of myeloid-derived OCs [7, 37, 54] as evidenced by the lack of OC differentiation in RANK^(−/−)^ mice [60]. Our study provides evidence that FHb can attenuate RANKL-induced RANK expression in RAW264.7 cells suggesting a possible mechanism by which FHb impairs OC differentiation from macrophages.

Osteoprotegerin (OPG) is a decoy receptor for RANKL and plays a regulatory role in bone resorption by inhibiting OC function [61]. As a dimer, OPG competes with RANK for RANKL binding and effectively inhibits RANK-RANKL interaction [62]. Here, by demonstrating the decrease of RANKL interaction with RANK in RAW264.7 cells, we provide an additional potential explanation for the involvement of FHb in the impairment of OC differentiation. This observation was corroborated by our test tube experiments, which support the hypothesis that FHb directly hinders RANK-RANKL interaction. Our results suggest that FHb inhibits OC formation in an OPG-like manner by directly inhibiting RANK-RANKL interaction. The exact mechanism of such interaction requires further investigations.

## 5. Conclusions

**Figure 8:**
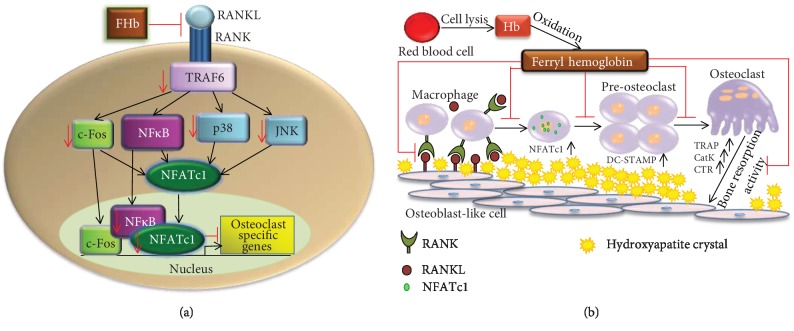
(a) FHb inhibits RANK-RANKL interaction and blocks osteoclastogenic signalization pathways downstream of RANK such as tumor necrosis factor (TNF) receptor-associated factor 6 (TRAF6), c-Jun N-terminal kinase (JNK), p38, c-Fos, and receptor activator of nuclear factor-kappa B (NF*κ*B), thereby preventing the expression and nuclear translocation of nuclear factor of activated T-cells, cytoplasmic 1 (NFATc1), and subsequent expression of OC-specific genes. (b) FHb, which is abundantly present in hemorrhagic calcified lesions, impedes the formation of OCs from macrophages disturbing OC bone resorption activity by downregulating OC-specific gene expression such as NFATc1, DC-STAMP, TRAP, CatK, and CTR. During vascular calcification, smooth muscle cells in the vessel walls undergo osteochondrogenic reprogramming and produce RANKL, which initiates OC formation from macrophages as a potential compensatory mechanism to remove calcium deposits. However, the presence of FHb in hemorrhagic atheromas might create a unique microenvironment where OLCs-mediated resorption of calcium deposits is impaired, thereby blocking the endogenous calcium resorption capability in the vasculature.

In summary, we provide evidence for the involvement of FHb in the inhibition of osteoclastogenesis (Figure 8). This effect of FHb suggests that the presence of FHb in hemorrhagic atheromas might create a unique microenvironment where OLC-mediated resorption of calcium deposits is impaired that blocks the endogenous calcium resorption capability in the vasculature.

## Data Availability

The data that support the findings of this study are available from the corresponding author upon reasonable request.

## Supplementary Materials

Supplementary Materials Supplementary Figure 1: RAW264.7 cells were cultured in control growth media or in osteoclastogenic media (control growth media supplemented with 50 ng/mL RANKL) in the absence or presence of FHb (10 *μ*mol/L heme group) for 4 days. (A) Cell proliferation and viability were analyzed with MTT 38 assay at various time points (one to four days). (B) Caspase-3 cleavage was analyzed after 4 days with immunoblot. Supplementary Figure 2: Recombinant 6 × His-tagged RANKL was expressed in E. coli Rosetta 2. (A) Purity of recombinant RANKL was analyzed by Coomassie-staining (left panel) and immunoblot (right panel). (B) RAW264.7 cells were cultured in control growth media or in osteoclastogenic medium (using 6 × His-tagged RANKL) with or without FHb (10 *μ*mol/L heme group) for 5 days. Biological activity of 6 × His-tagged RANKL was verified by TRAP staining. RANK was immunoprecipitated from RAW264.7 cell lysates and coincubated with His-tagged recombinant RANKL (1 *μ*g) in the presence of absence of FHb. The association of recombinant RANKL with RANK was analyzed by immunoblot.
