# Supplementary figures and images for: Ferryl Hemoglobin Inhibits Osteoclastic Differentiation of Macrophages in Hemorrhaged Atherosclerotic Plaques
Source: Oxid Med Cell Longev. 2020 Feb 27;2020:3721383. doi: 10.1155/2020/3721383 (PMC7063196; doi:10.1155/2020/3721383)

Supplementary figure 1

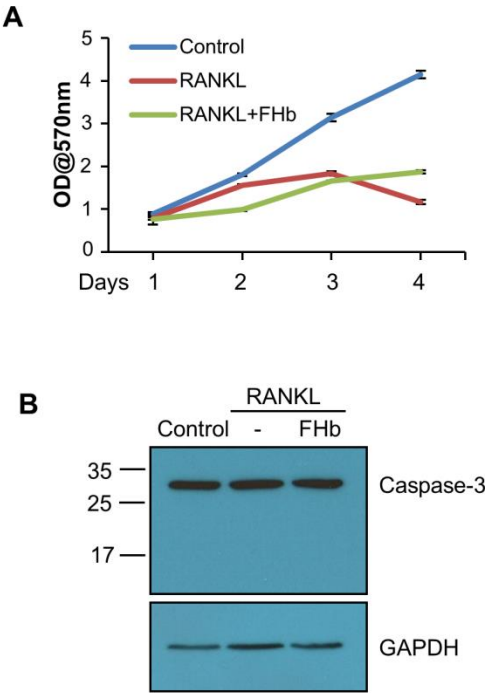

Supplementary figure 2

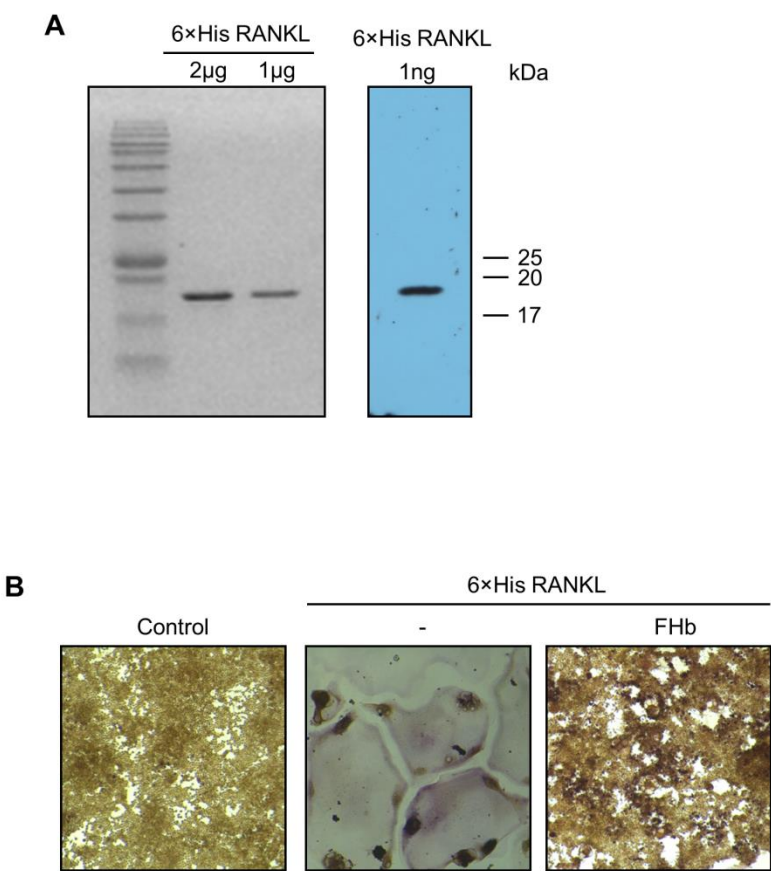

Supplement: Supplementary Materials — Supplementary Figure 1: RAW264.7 cells were cultured in control growth media or in osteoclastogenic media (control growth media supplemented with 50 ng/mL RANKL) in the absence or presence of FHb (10 μmol/L heme group) for 4 days. (A) Cell proliferation and viability were analyzed with MTT 38 assay at various time points (one to four days). (B) Caspase-3 cleavage was analyzed after 4 days with immunoblot. Supplementary Figure 2: Recombinant 6 × His-tagged RANKL was expressed in E. coli Rosetta 2. (A) Purity of recombinant RANKL was analyzed by Coomassie-staining (left panel) and immunoblot (right panel). (B) RAW264.7 cells were cultured in control growth media or in osteoclastogenic medium (using 6 × His-tagged RANKL) with or without FHb (10 μmol/L heme group) for 5 days. Biological activity of 6 × His-tagged RANKL was verified by TRAP staining. RANK was immunoprecipitated from RAW264.7 cell lysates and coincubated with His-tagged recombinant RANKL (1 μg) in the presence of absence of FHb. The association of recombinant RANKL with RANK was analyzed by immunoblot. [file 3721383.f1.pdf]
